# Persistent dyspnea after COVID-19 is not related to cardiopulmonary impairment; a cross-sectional study of persistently dyspneic COVID-19, non-dyspneic COVID-19 and controls

**DOI:** 10.3389/fphys.2022.917886

**Published:** 2022-07-06

**Authors:** Rhys I. Beaudry, Andrew R. Brotto, Rhea A. Varughese, Stephanie de Waal, Desi P. Fuhr, Ronald W. Damant, Giovanni Ferrara, Grace Y. Lam, Maeve P. Smith, Michael K. Stickland

**Affiliations:** ^1^ Division of Pulmonary Medicine, Department of Medicine, Faculty of Medicine and Dentistry, University of Alberta, Edmonton, AB, Canada; ^2^ G.F. MacDonald Centre for Lung Health, Covenant Health, Edmonton, AB, Canada

**Keywords:** DLCO, long-covid, pulmonary function, pulmonary vascular, VO_2_, maximal oxygen consumption

## Abstract

**Background:** Up to 53% of individuals who had mild COVID-19 experience symptoms for >3-month following infection (Long-CoV). Dyspnea is reported in 60% of Long-CoV cases and may be secondary to impaired exercise capacity (VO_2peak_) as a result of pulmonary, pulmonary vascular, or cardiac insult. This study examined whether cardiopulmonary mechanisms could explain exertional dyspnea in Long-CoV.

**Methods:** A cross-sectional study of participants with Long-CoV (n = 28, age 40 ± 11 years, 214 ± 85 days post-infection) and age- sex- and body mass index-matched COVID-19 naïve controls (Con, n = 24, age 41 ± 12 years) and participants fully recovered from COVID-19 (ns-CoV, n = 14, age 37 ± 9 years, 198 ± 89 days post-infection) was conducted. Participants self-reported symptoms and baseline dyspnea (modified Medical Research Council, mMRC, dyspnea grade), then underwent a comprehensive pulmonary function test, cardiopulmonary exercise test, exercise pulmonary diffusing capacity measurement, and rest and exercise echocardiography.

**Results:** VO_2peak_, pulmonary function and cardiac/pulmonary vascular parameters were not impaired in Long- or ns-CoV compared to normative values (VO_2peak_: 106 ± 25 and 107 ± 25%_predicted_, respectively) and cardiopulmonary responses to exercise were otherwise normal. When Long-CoV were stratified by clinical dyspnea severity (mMRC = 0 vs mMRC≥1), there were no between-group differences in VO_2peak_. During submaximal exercise, dyspnea and ventilation were increased in the mMRC≥1 group, despite normal operating lung volumes, arterial saturation, diffusing capacity and indicators of pulmonary vascular pressures.

**Interpretation:** Persistent dyspnea after COVID-19 was not associated with overt cardiopulmonary impairment or exercise intolerance. Interventions focusing on dyspnea management may be appropriate for Long-CoV patients who report dyspnea without cardiopulmonary impairment.

## Introduction

Up to 87% of individuals hospitalized as a result of COVID-19 and 53% of individuals with mild initial illness experience symptoms 3-month after infection; these individuals are colloquially known as COVID Long-Haulers (Long-CoV) (Carfì et al., 2020; Petersen et al., 2020; World Health Organization, 2021). Dyspnea is reported in approximately 60% of Long-CoV cases; little evidence is available directly linking persistent dyspnea after COVID-19 to known physiological mechanisms for increased dyspnea sensation (Davis et al., 2021).

Long-CoV is predominately thought to be due to lingering symptoms from impairments incurred during active infection. However, emerging evidence suggests a dissociation between pathophysiology and symptom burden, particularly in non-severe COVID where initial cardiopulmonary damage may be trivial. Lam et al. (2021) reported a Long-CoV phenotype characterized by persistent dyspnea, impaired 6-min walk distance and reduced health-related quality of life, but without accompanying pulmonary function abnormalities or increased neurologic, musculoskeletal or fatigue symptoms which could contribute to dyspnea and reduced cardiopulmonary fitness. Conversely, in a study of 103 hospitalized patients with overt cardiopulmonary impairment, Lerum et al. (2021) report that at 3-month follow-up, forced vital capacity (FVC), forced expiratory volume in 1 s (FEV_1_), diffusing capacity for carbon monoxide (DLCO), and ground glass opacities and parenchymal bands on chest CT improved, independent of improvements in 6-min walk distance or dyspnea score. These findings suggest that while cardiopulmonary function and symptoms may improve concomitantly, cardiopulmonary improvement may not relate to symptom improvement. In a large cohort (n = 26,823), Long-CoV symptom burden had a stronger association with belief in having had COVID-19, rather than serology confirmed history of infection, suggesting that symptoms may be falsely attributed to COVID-19 and may be a result of other or pre-existing conditions (Matta et al., 2022). Indeed, while several studies have reported cardiopulmonary fitness in Long-CoV, these patient samples contain a high proportion of participants with substantial smoking history, obesity, and cardiovascular comorbidities, all of which confound the ability to isolate and investigate COVID-19-related dyspnea (Clavario et al., 2020; Jahn et al., 2021; Motiejunaite et al., 2021). Though these study samples are consistent with Long-CoV risk factors, it remains unclear whether cardiopulmonary findings are incidental or due to COVID-19.

Here, we sought to examine the cardiorespiratory responses to exercise in isolated Long-CoV and determine whether persistent symptoms/dyspnea are associated with altered cardiorespiratory function. In other pathologies, such as chronic obstructive pulmonary disease (COPD), interstitial lung disease (ILD), and pulmonary hypertension (PH), exertional dyspnea can be due to the inability to maintain appropriate gas exchange, an exaggerated ventilatory response to exercise, elevated operating lung volumes (i.e., dynamic hyperinflation), and/or elevated pulmonary vascular pressures, all of which have detrimental impacts on exercise capacity (VO_2peak_) (Arena and Sietsema, 2011; Faisal et al., 2015; Obokata et al., 2018). We hypothesized that: VO_2peak_ would be impaired in Long-CoV; and that reduced VO_2peak_ would be associated with 1) elevated exertional dyspnea, 2) a greater ventilatory response to exercise, 3) altered breathing mechanics, or 4) reduced DLCO.

## Materials and methods

### Ethical approval

This study was approved by the University of Alberta Human Research Ethics Board (Pro00107436), registered as a clinical trial (NCT04732663) and conducted in accordance with the Declaration of Helsinki. All participants provided written, informed consent prior to enrollment.

### Design

A cross-sectional study design was used to compare participants with self-reported Long-CoV to participants whom were no-longer symptomatic from COVID-19 (ns-CoV) and COVID-19 naïve controls (Con).

### Participants

Long-CoV participants were at least 12-week from first molecular test positivity (mean time from test positivity to first research visit = 219 ± 82 days) and experiencing at least one persistent symptom (ageusia, anosmia, cough, diarrhea, dyspnea, fatigue, fever, headache, muscle pain) at the time of testing (World Health Organization, 2021). Participants were recruited and tested between March and August 2021 (latest possible infection, April 2021); no participants had received a vaccination against COVID-19 prior to their infection. Participants were excluded for: 1) diagnosis of PH predating COVID-19; 2) absolute contraindication to exercise testing or orthopedic limitation; 3) age <18 or >65 years; and 4) body mass index (BMI) > 30 kg/m^2^ to limit confounding obesity effects on cardiopulmonary function. Participants were recruited from the University of Alberta/Kaye Edmonton Post-COVID clinic. Con and ns-CoV participants were recruited by word of mouth from the greater Edmonton area and matched to Long-CoV participants for age, sex and BMI.

Participants were then stratified by modified Medical Research Council (mMRC) dyspnea grade to dyspneic-CoV (mMRC≥1) and retrospectively matched to non-dyspneic-CoV (mMRC = 0 from ns- or Long-CoV), and controls for sex, age and BMI to explore relationships between impaired cardiorespiratory physiology and persistent dyspnea.

### Procedures

Participants reported to the laboratory at the University of Alberta—Edmonton, Canada—for three visits.

### Visit 1

Participants first completed a medical history and the mMRC Dyspnea Scale, Post-COVID Functional Scale (PCFS; 0, no functional limitations; 1, negligible functional limitations; 2, slight functional limitations; 3, moderate functional limitations; 4, severe functional limitations) and EuroQoL-5D-5L Visual Analog Scale (EQ-5D VAS, scored 0–100; *0, the worst health you can imagine*; *100, the best health you can imagine*) questionnaires (Mahler and Wells, 1988; Herdman et al., 2011; Klok et al., 2020).

A pulmonary function test (PFT) was conducted in accordance with American Thoracic Society/European Respiratory Society guidelines, results are reported as percent of predicted (Graham et al., 2017; Graham et al., 2019; Hall et al., 2021). Participants then underwent an incremental cardiopulmonary exercise test as previously described (Phillips et al., 2021). Briefly, an incremental test was conducted on an electronically braked cycle ergometer (Ergoselect II 1200, Ergoline, Blitz, Germany) starting at 0 W (unloaded cycling) and progressing by 20 W every 2 min. Ratings of perceived exertion (RPE) for dyspnea and leg discomfort (modified Borg scale, 1–10) (Borg, 1982) and inspiratory capacity (IC) maneuvers were conducted every 2-min. A good quality test was based on attainment of three of the four following criteria: 1) volitional exhaustion; 2) respiratory exchange ratio (RER) greater than 1.10; 3) maximal heart rate within 10 beats per minute (bpm) of age predicted maximum heart rate (208—[0.7 x age]) (Tanaka et al., 2001); 4) increase in oxygen consumption <100 ml/min with an increase in power output (Stickland et al., 2012). Expired gas was analyzed using a metabolic measurement system (Encore229 Vmax, SensorMedics, Yorba Linda, United States); arterial oxygen saturation (SpO_2_) was estimated using finger pulse oximetry (N-595 Oximax, Nellcor, Boulder, United States), heart rate was measured using a 12-lead electrocardiogram (CardioSoft, GE Medical Systems, Milwaukee, United States) and blood pressure was taken by manual auscultation. Measures were continuously recorded and averaged in 30-s blocks; VO_2 peak_ values were compared using prediction equations from population-based samples (Neder et al., 1999; Lewthwaite et al., 2020).

### Visit 2

The multiple fraction of inspired oxygen (F_I_O_2_)-DLCO technique was used to measure DLCO, pulmonary capillary blood volume (V_Cap_) and membrane diffusing capacity (D_M_) at rest, 40 W, and 80% of peak power, as previously described (Tedjasaputra et al., 2017; Tedjasaputra et al., 2018). Briefly, DLCO measurements were performed using a 6-s breath-hold; participants waited a minimum of 4-min between breath-holds at rest, and 2-min between breath-holds during exercise. Participants cycled continuously for the 40 W stage, and for 2-min at 80% of their maximum prior to conducting each breath-hold and returning to lower intensity cycling for active recovery.

### Visit 3

A brief echocardiographic study was performed at rest and during cycling exercise at 40 W (Ergoselect 1200 Stress Echo Supine Ergometer, Blitz, Germany) in optimal echocardiography position (supine/tilted). Images were collected by a Canadian Registered Cardiac Sonographer using a commercially available ultrasound device (Vivid Q, GE Healthcare, Fairfield, United States). To reduce bias, blinded analysis was performed using the manual measurement tool of a commercially available analysis software (Us2. ai, Singapore, Singapore) by certified imaging specialists in accordance with American Society of Echocardiography guidelines and independently verified (Mitchell et al., 2019).

### Protocol modification

Ongoing data analysis of the first n = 22 Long-CoV participants versus n = 9 ns-CoV and n = 16 Con yielded no significant differences, or trends for differences in exercising DLCO, V_Cap_, D_M_, or echocardiography derived measures. Therefore, the investigators ceased Visit 2 and 3 testing in the remaining n = 6 Long-CoV, n = 5 ns-CoV and n = 8 Con. A sensitivity analysis of VO_2peak_ (percent predicted) was conducted and no differences were found in each group between those completing all visits, versus those who completed only Visit 1.

### Study size

A 3.5 ml/kg/min reduction in VO_2peak_ is associated with a 12% and 17% decrease in survival in men and women respectively (Myers et al., 2002; Gulati et al., 2003). It was calculated that sixteen participants in each group would be sufficient to detect a 3.5 ± 3.5 ml/kg/min mean difference in VO_2peak_ between groups (*α* = 0.05, β = 0.80).

### Statistics

Data are presented as mean ± standard deviation unless otherwise stated. Statistical significance was set *a priori* at *p* < 0.05. Participant characteristics, pulmonary function, highest equivalent work rate and peak CPET data were compared between groups by one-way analysis of variance (ANOVA). Shapiro-Wilk tests were used to test normality in all outcomes prior to ANOVA. Bonferroni T-tests were used when ANOVA revealed a main effect and/or interaction. All statistical analyses were performed using IBM SPSS Statistics 24 (IBM Corporation, Armonk, United States).

## Results

### Participants

**FIGURE 1:**
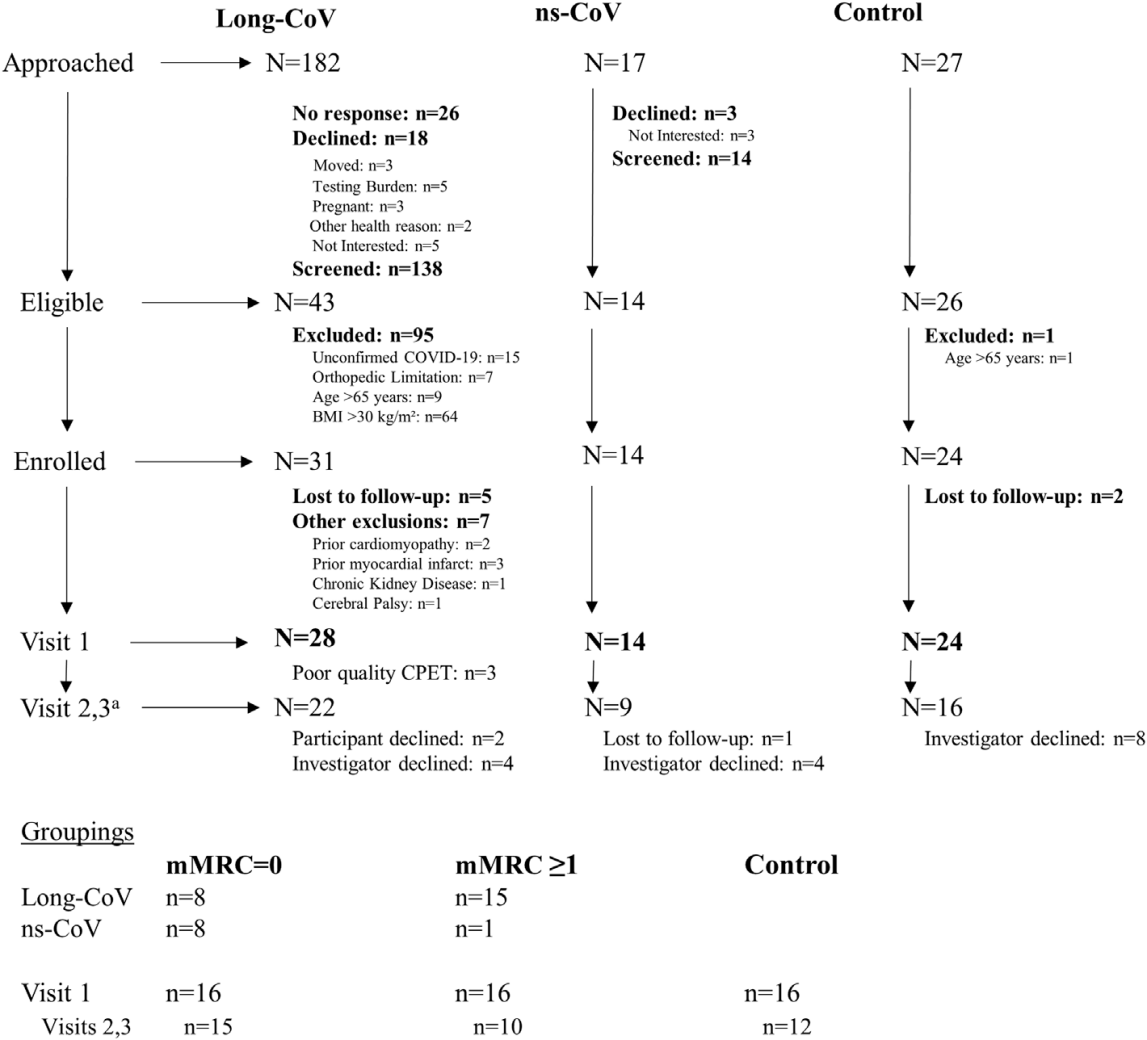
Participant recruitment, eligibility, enrollment and testing. ^a^Ongoing analysis showed no evidence of differences in exercising DLCO or echocardiography derived measurements. Therefore, investigators declined testing participants on Visits 2 and 3 as testing was not justifiable.

Recruitment, eligibility, enrollment and testing details are displayed in Figure 1. Participant characteristics are displayed in Tables 1, 2. Data are reported for twenty-eight Long-CoV, all of whom were seeking medical care for persistent symptoms. Participants were well matched between Long-CoV and Con for age, sex and BMI; fewer ns-CoV (n = 14) were recruited, however, the participant characteristics of the sample were not different from Long-CoV or Con (n = 24). Self-reported symptoms during acute infection are reported in Table 1; notably, all participants with a history of COVID-19 experienced symptoms during active infection. Fifty-eight percent of Long-CoV participants reported persistent dyspnea. Self-reported overall health (EQ-5D) and physical functioning (PCFS) were reduced in Long-CoV relative to ns-CoV and Con (Table 1). Participant characteristics after grouping by mMRC dyspnea grade are displayed in Table 2. Dyspneic-CoV participants were free of cardiovascular comorbidities and conditions that could provide an alternative explanation for dyspnea. Self-reported overall health (EQ-5D) and physical functioning (PCFS) were reduced in dyspneic-CoV relative to mMRC = 0 and Con groups.

**TABLE 1:** Participant characteristics, symptoms during acute infection, and time since molecular confirmed positive COVID-19 test for Long-CoV, no-longer symptomatic (ns)-CoV and Controls. BMI, body mass index; BSA, body surface area; PCFS, Post-COVID Functional Scale; EQ-5D VAS, EuroQol 5-Dimension visual analog scale.

Self-reported symptom groupings		Long-CoV	Ns-CoV	Control
Sex (F/M), N		20/8	10/4	17/7
Age (years)		40 (11)	37 (9)	41 (12)
Height (cm)		168 (8)	170 (8)	169 (8)
BMI (kg/m^2^)		24.7 (3.1)	23.0 (3.1)	23.6 (3.2)
BSA (m^2^)		1.81 (0.19)	1.77 (0.20)	1.77 (0.19)
Smoking History (n)		4	2	3
Pre-existing airway obstruction (n)		5	1	1
Cardiovascular Comorbidity (n)		3	2	2
Acute COVID	Symptom Length (days)	20 (18)	19 (17)	-
	Hospital Admission (n, %)	4 (13%)	3 (21%)	-
	Emergency Visit (n, %)	5 (17%)	2 (14%)	-
	Dyspnea (n, %)	24 (80%)	13 (93%)	-
	Fatigue (n, %)	26 (87%)	7 (50%)	-
	Fever (n, %)	20 (67%)	10 (71%)	-
	Anosmia (n, %)	19 (63%)	8 (57%)	-
	Loss of Taste (n, %)	18 (60%)	8 (57%)	-
	Muscle Pain (n, %)	16 (53%)	11 (79%)	-
	Headaches (n, %)	20 (67%)	8 (57%)	-
	Cough (n, %)	19 (63%)	8 (57%)	-
	Diarrhea (n, %)	7 (23%)	1 (7%)	-
Time post infection	Days post positive test	214 (85)	198 (89)	-
Quality of Life	PCFS	1 (1)^†‡^	0 (0)	0 (0)
	EQ-5D (VAS)	71 (16)^†‡^	82 (12)	89 (9)

†: *p* < 0.05 vs. Con; ‡: *p* < 0.05 vs. ns-CoV.

**TABLE 2:** Participant characteristics, symptoms during acute infection, and time since molecular confirmed positive COVID-19 test for COVID-19 groups after stratification by mMRC dyspnea grade (0 = normal, ≥1 = dyspneic) and Controls. BMI, body mass index; BSA, body surface area; PCFS, Post-COVID Functional Scale; EQ-5D VAS, EuroQol 5-Dimension visual analog scale.

COVID split by mMRC		mMRC≥1	mMRC = 0	Control
Sex (F/M), N		13/3	13/3	13/3
Age (years)		39 (11)	39 (11)	41 (11)
Height (cm)		166 (6)	170 (9)	167 (6)
BMI (kg/m^2^)		25.0 (3.2)	23.7 (3.3)	23.6 (3.3)
BSA (m^2^)		1.78 (0.16)	1.79 (0.21)	1.75 (0.15)
Smoking History (n, %)		3 (23)	2 (12)	3 (23)
Acute COVID	Symptom Length (days)	23 (22)	17 (15)	-
	Hospitalization (n, %)	4 (24%)	2 (12%)	-
	Emergency Visit (n, %)	4 (24%)	2 (12%)	-
	Dyspnea (n, %)	16 (94%)	9 (53%)	-
	Fatigue (n, %)	13 (76%)	15 (88%)	-
	Fever (n, %)	12 (71%)	12 (71%)	-
	Anosmia (n, %)	12 (71%)	10 (59%)	-
	Ageusia (n, %)	12 (71%)	10 (59%)	-
	Muscle Pain (n, %)	11 (65%)	8 (47%)	-
	Headaches (n, %)	12 (71%)	10 (59%)	-
	Cough (n, %)	13 (76%)	8 (47%)	-
	Diarrhea (n, %)	4 (24%)	1 (6%)	-
Time post infection	Days post positive test	202 (74)	215 (104)	-
Quality of Life	PCFS	2 (1)^†‡^	0 (0)	0 (0)
	EQ-5D VAS	66 (19)^†‡^	83 (9)	89 (9)

†: *p* < 0.05 vs. Con; ‡: *p* < 0.05 vs. mMRC = 0.

### Pulmonary function testing

No differences were found between Long-CoV, ns-CoV, or Con for resting pulmonary function and means were all within normal limits (Table 3). When participants were split by mMRC grade, no differences in resting pulmonary function were found between groups, and means were within normal limits (Table 4). Three mMRC ≥1 participants, 4 mMRC = 0 participants, and 1 Con participant had an FEV_1_:FVC ratio below the LLN; 2 participants in the mMRC ≥1 group had a TLC below the LLN; and 1 participant in each of the mMRC = 0 and ≥1 groups had a DLCO below the LLN.

**TABLE 3:** Resting pulmonary function (pulmonary function test, PFT) and submaximal and peak exercise data for Long-CoV, no-longer symptomatic (ns)-CoV and Controls.

Self-reported symptom groupings			Long-CoV	Ns-CoV	Con
PFT	FVC	l	4.25 (0.76)	4.19 (1.07)	4.36 (0.84)
		% pred	104 (11)	99 (16)	105 (8)
		n < LLN	0	1	0
	FEV_1_	l	3.29 (0.63)	3.29 (0.72)	3.49 (0.70)
		% pred	98 (11)	94 (13)	102 (9)
		n < LLN	1	1	0
	FEV_1_:FVC	ratio	0.78 (0.08)	0.79 (0.08)	0.80 (0.05)
		% pred	94 (8)	96 (9)	97 (6)
		n < LLN	4	2	1
	IC	l	2.93 (0.55)	2.73 (0.74)	3.10 (0.78)
		% pred	99 (13)	91 (18)	104 (15)
		n < LLN	0	3	0
	TLC	l	5.81 (1.02)	5.67 (1.25)	5.85 (1.06)
		% pred	100 (11)	95 (14)	99 (8)
		n < LLN	1	1	0
	RV	l	1.48 (0.43)	1.45 (0.37)	1.44 (0.38)
		% pred	102 (23)	104 (29)	100 (26)
	DLCO	ml/min/mmHg	25.4 (5.2)	26.8 (8.8)	25.9 (5.0)
		n < LLN	3	1	1
		Adj. VA	25.1 (5.7)	26.5 (10.0)	25.6 (5.5)
		% pred	104 (15)	106 (21)	105 (13)
Peak	VO_2_	% pred	106 (25)^†^	107 (25)	129 (25)
		n < LLN	3	1	0
		ml/kg/min	32 (9)^†^	35 (10)	40 (9)
	VE	% pred	123 (30)	121 (24)	133 (32)
	RER	ratio	1.21 (0.07)	1.21 (0.07)	1.22 (0.06)
	HR	bpm	175 (14)	179 (8)	178 (13)
		% pred	102 (9)	102 (5)	104 (6)
	RPE	Legs	8 (2)	9 (2)	9 (1)
		Dyspnea	7 (2)	8 (2)	8 (2)
	SpO_2_	%, at peak	96 (3)	95 (3)	96 (3)
	BP	Systolic, mmHg	153 (18)	155 (10)	159 (22)
		Diastolic, mmHg	72 (10)	71 (10)	74 (11)
	Hb	g/dl, at peak	14.8 (1.5)	15.2 (1.7)	15.4 (1.4)
Sub-max	Multiple F_I_O_2_-DLCO	V_Cap at 40w_, ml	81 (27)	83 (38)	69 (12)
		V_Cap adj. VA_, ml	76 (17)	80 (33)	69 (12)
		D_M_, ml/min/mmHg	76 (38)	87 (49)	82 (29)
	Highest Equiv. Work Rate	Power (w)	80	80	80
		VE (L/min)	39 (8)^†^	36 (6)	34 (5)
		IRV (%TLC)	22 (8)	22 (4)	25 (7)
		Dyspnea (Borg)	3 (1)	2 (1)	2 (1)

FVC, forced vital capacity; FEV_1_, forced expiratory volume in one second; IC, inspiratory capacity; TLC, total lung capacity; RV, residual volume; VO_2_, oxygen uptake; VE, minute ventilation; RER, respiratory exchange ratio; HR, heart rate; RPE, modified Borg (0–10) rating of perceived exertion; SpO_2_, arterial oxygen saturation; BP, blood pressure; Hb, hemoglobin; LLN, lower limit of normal; F_I_O_2_-DLCO, multiple fraction of inspired oxygen diffusing capacity for carbon monoxide; VA, alveolar volume; V_Cap_, pulmonary capillary blood volume; D_M_, diffusing membrane capacity; IRV, inspiratory reserve volume. †: *p* < 0.05 vs. Con; ‡: *p* < 0.05 vs. ns-CoV.

**TABLE 4:** Resting pulmonary function (pulmonary function test, PFT) and submaximal and peak exercise data for mMRC≥1 (dyspneic-CoV), mMRC = 0 and Controls.

COVID split by mMRC			mMRC≥1	mMRC = 0	Con
PFT	FVC	l	3.88 (0.51)	4.44 (0.95)	4.16 (0.63)
		% pred	101 (9)	106 (14)	104 (9)
		n < LLN	0	0	0
	FEV_1_	l	3.06 (0.55)	3.40 (0.78)	3.29 (0.48)
		% pred	96 (9)	98 (12)	100 (9)
		n < LLN	0	0	0
	FEV_1_:FVC	ratio	0.79 (0.09)	0.77 (0.06)	0.79 (0.06)
		% pred	95 (9)	93 (7)	96 (7)
		n < LLN	1	1	1
	IC	l	2.73 (0.53)	2.99 (0.61)	2.89 (0.59)
		%pred	97 (12)	100 (14)	100 (14)
		n < LLN	0	0	0
	TLC	l	5.32 (0.75)	6.03 (1.13)	5.63 (0.85)
		% pred	95 (10)	102 (12)	99 (9)
		n < LLN	2	0	0
	RV	l	1.35 (0.45)	1.49 (0.38)	1.43 (0.42)
		% pred	96 (24)	102 (23)	101 (28)
	DLCO	ml/min/mmHg	24.1 (3.9)	26.6 (8.7)	24.9 (4.3)
		n < LLN	1	1	0
		Adj. VA	23.4 (4.0)	26.8 (9.5)	24.6 (4.7)
		% pred	103 (14)	106 (17)	105 (13)
Peak	VO_2_	% pred	98 (20)^†^	113 (26)	124 (26)
		n < LLN	2	1	0
		ml/kg/min	29.0 (6.8)^†^	34.8 (8.6)	37.9 (9.6)
	VE	% pred	118 (30)	121 (22)	129 (33)
	RER	ratio	1.21 (0.07)	1.19 (0.07)	1.22 (0.06)
	HR	bpm	174 (11)	177 (13)	177 (14)
		% pred	100 (5)	103 (9)	103 (5)
	RPE	Legs	8 (2)	9 (2)	9 (1)
		Dyspnea	7 (3)	7 (2)	8 (1)
	SpO_2_	%, at peak	96 (4)	96 (4)	96 (3)
	BP	Systolic, mmHg	150 (18)	154 (14)	159 (24)
		Diastolic, mmHg	70 (10)	72 (10)	74 (11)
	Hb	g/dl, at peak	14.9 (1.4)	14.8 (1.9)	15.4 (1.6)
Sub-max	Multiple F_I_O_2_-DLCO	V_Cap at 40w_, ml	76 (22)	87 (38)	70 (13)
		V_Cap adj. VA_, ml	73 (13)	81 (29)	69 (13)
		D_M_, ml/min/mmHg	70 (35)	76 (38)	76 (22)
	Highest Equiv. Work Rate	Power (w)	80	80	80
		VE (L/min)	40 (9)^†^	38 (5)^†^	34 (5)
		IRV (%TLC)	22 (8)	22 (5)	24 (7)
		Dyspnea (Borg)	3 (2)^†‡^	2 (1)	2 (1)

FVC, forced vital capacity; FEV_1_, forced expiratory volume in one second; IC, inspiratory capacity; TLC, total lung capacity; RV, residual volume; VO_2_, oxygen uptake; VE, minute ventilation; RER, respiratory exchange ratio; HR, heart rate; RPE, modified Borg (0–10) rating of perceived exertion; SpO_2_, arterial oxygen saturation; BP, blood pressure; Hb, hemoglobin; LLN, lower limit of normal; F_I_O_2_-DLCO, multiple fraction of inspired oxygen diffusing capacity for carbon monoxide; VA, alveolar volume; V_Cap_, pulmonary capillary blood volume; D_M_, diffusing membrane capacity; IRV, inspiratory reserve volume. †: *p* < 0.05 vs. Con; ‡: *p* < 0.05 vs. mMRC = 0.

### Cardiopulmonary exercise testing

**FIGURE 2:**
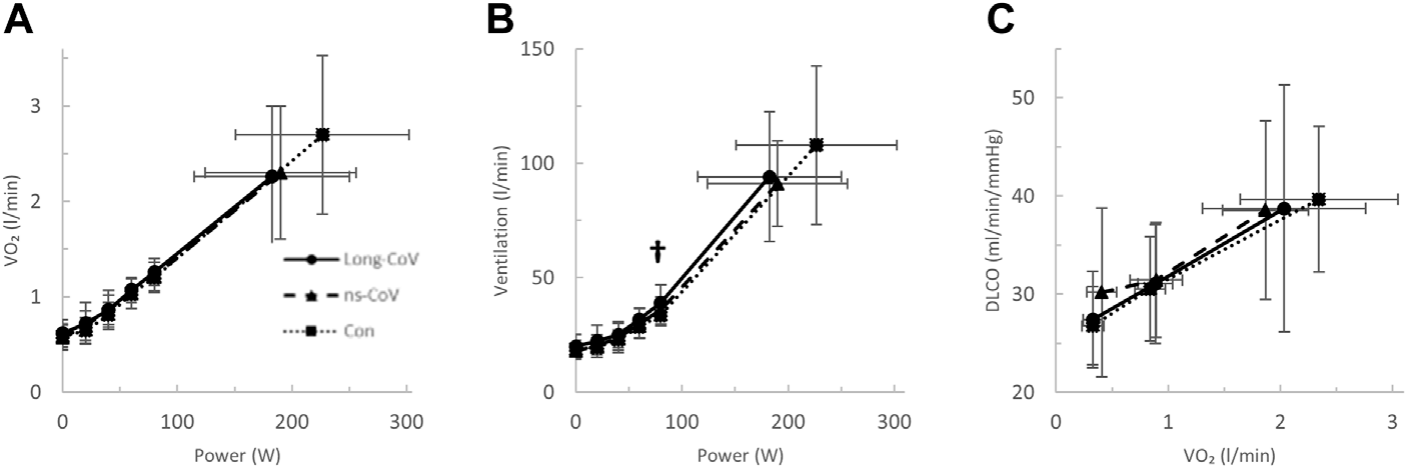
The **(A)** oxygen uptake (VO_2_)- and **(B)** ventilation (VE)-power relationships and the diffusing capacity for carbon monoxide (DLCO)-VO_2_ relationships in Long-CoV (circles, solid line), no-longer symptomatic (ns)-CoV (triangles, dashed line) and Con (squares, dotted line). † = *p* < 0.05 Long-CoV vs Con.

Long-CoV and ns-CoV had normal VO_2peak_ (mean 106 ± 25 and 107 ± 25%_predicted_, respectively), but significantly lower VO_2peak_ than Con (130%_predicted_) (Table 3; Figure 2A). Submaximal oxygen uptake and DLCO responses to exercise were similar for all groups (Figures 2A,C). Arterial saturation (Table 3) and the components of diffusing, D_M_ and V_Cap_ (Table 3), were not different between groups, indicating normal gas exchange.

**FIGURE 3:**
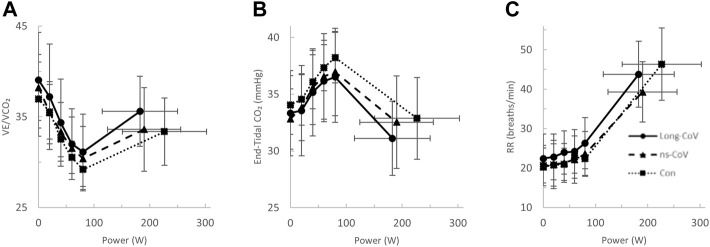
The **(A)** ventilatory efficiency (VE/VCO_2_)-, **(B)** end-tidal CO_2_−, and **(C)** respiratory rate (RR)-power relationships in Long-CoV (circles, solid line), no-longer symptomatic (ns)-CoV (triangles, dashed line) and Con (squares, dotted line). No significant differences.

**FIGURE 4:**
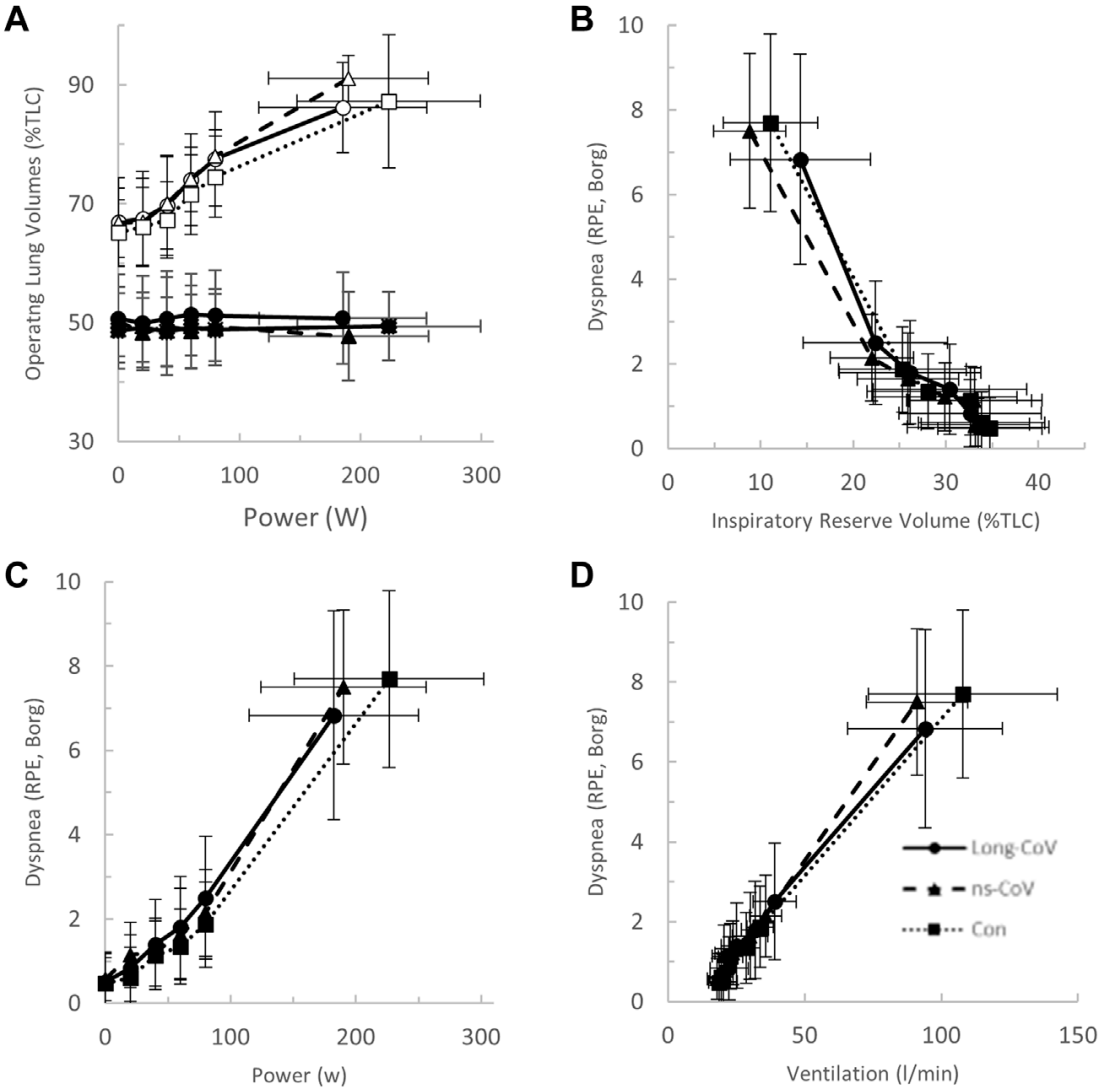
**(A)** The operating lung volume-power (open, end-inspiratory lung volume, closed, end-expiratory lung volume); **(B)** dyspnea-inspiratory reserve volume (as percent of total lung capacity, TLC); **(C)** dyspnea-power (Borg rating of perceived exertion, RPE); and **(D)** dyspnea-ventilation relationships in Long-CoV (circles, solid line), no-longer symptomatic (ns)-CoV (triangles, dashed line) and Con (squares, dotted line). No significant differences.

At the highest equivalent work rate (80 W), ventilation (VE, Long-CoV; 39 ± 8 vs. Con; 34 ± 5 L/min, *p* = 0.01) and the VE/VCO_2_ nadir were higher (i.e., less efficient ventilation) in Long-CoV relative to Con (Long-CoV; 29.8 ± 3.4 vs Con; 27.0 ± 3.6, *p* = 0.05), and respiratory rate (Long-CoV; 26 ± 6 vs. Con; 23 ± 5 breaths/minute, *p* = 0.07) trended to be higher, while end-tidal CO_2_ (P_ET_CO_2_) was not different (Long-CoV; 36.3 ± 4.0 vs. Con; 38.0 ± 2.4 mmHg, *p* = 0.22) (Figure 2B, Figures 3A–C). At the highest equivalent work rate (80 W), no differences were found between Long-CoV, ns-CoV and Con for operating lung volumes and inspiratory reserve volume (Figures 4A,B). Dyspnea relative to VE and dyspnea relative to inspiratory reserve volume with incremental exercise were not different across groups (Figures 4B,C).

**FIGURE 5:**
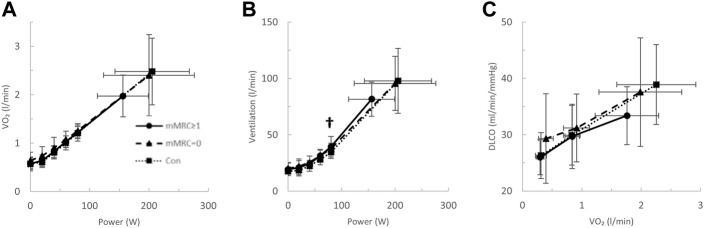
The **(A)** oxygen uptake (VO_2_)- and **(B)** ventilation (VE)-power relationships and the diffusing capacity for carbon monoxide (DLCO)-VO_2_ relationships in mMRC > 1 (dyspneic-CoV, circles, solid line), mMRC = 0 (triangles, dashed line) and Con (squares, dotted line). † = *p* < 0.05 mMRC≥1 vs Con.

**FIGURE 6:**
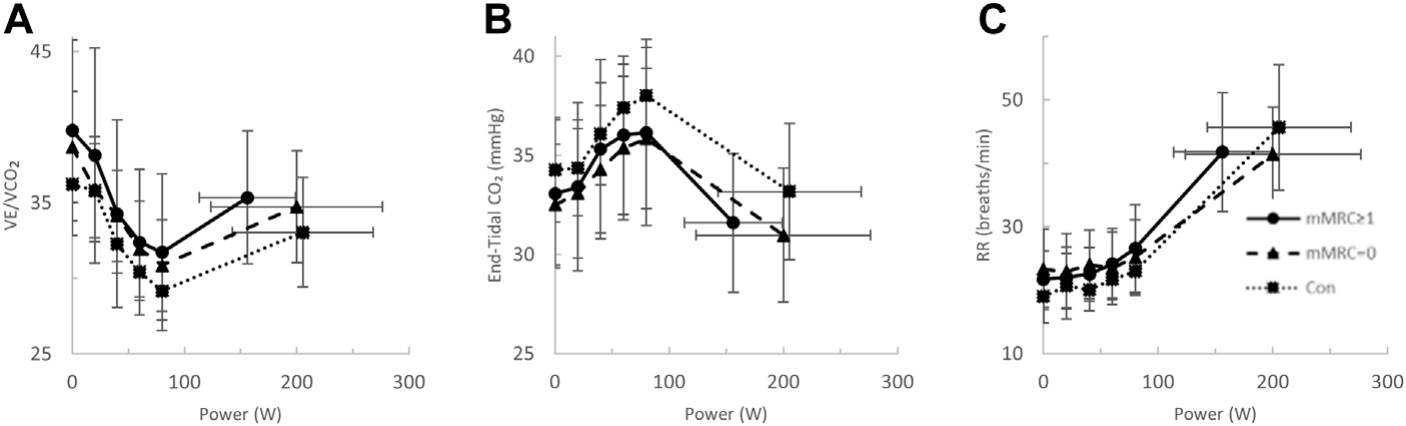
The ventilatory efficiency- (VE/VCO_2_, **(A)**, end-tidal CO_2_- **(B)** and respiratory rate- (RR, **(C)** power relationships in mMRC≥1 (dyspneic-CoV, circles, solid line), mMRC = 0 (triangles, dashed line) and Con (squares, dotted line). No significant differences.

**FIGURE 7:**
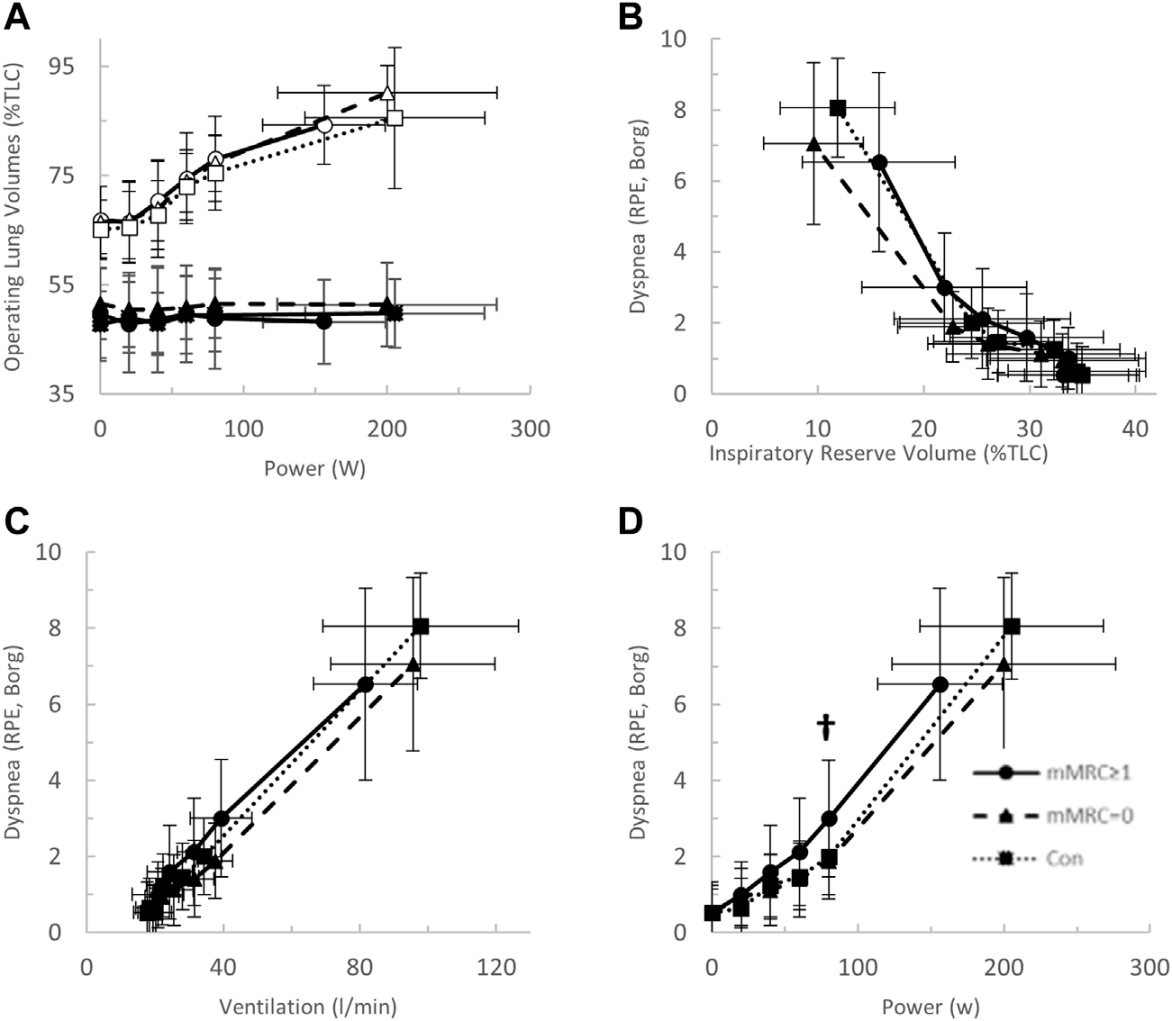
**(A)** The operating lung volume-power (open, end-inspiratory lung volume, closed, end-expiratory lung volume); **(B)** dyspnea-inspiratory reserve volume (as percent of total lung capacity, TLC); **(C)** dyspnea-power (Borg rating of perceived exertion, RPE); and **(D)** dyspnea-ventilation relationships in mMRC≥1 (dyspneic-CoV, circles, solid line), mMRC = 0 (triangles, dashed line) and Con (squares, dotted line). † = *p* < 0.05 mMRC≥1 vs Con.

After stratification by mMRC, no difference in VO_2peak_ was found between COVID-19 groups (Table 4; Figure 5A). Oxygen uptake, and DLCO responses to exercise were similar for all groups (Figures 5A,C). Arterial saturation (Table 4) and the components of diffusing capacity, D_M_ and V_Cap_ (Table 4), were not different between groups, indicating normal gas exchange. At the highest equivalent work rate (80 W), VE (dyspneic-CoV; 40 ± 9 vs. Con; 34 ± 5 L/min, *p* = 0.03) and dyspnea (dyspneic-CoV; 3 ± 2 vs. Con; 2 ± 1 Borg units, *p* = 0.04) were significantly higher for dyspneic-CoV as compared to Con, and respiratory rate (dyspneic-CoV; 27 ± 7 vs. Con; 23 ± 4 breaths/minute, *p* = 0.10) and the VE/VCO_2_ nadir (30.4 ± 4.0 vs 27.9 ± 2.3, *p* = 0.08) trended to be higher, while P_ET_CO_2_ (mMRC ≥1; 35.9 ± 4.7 vs. Con; 38.2 ± 2.4 mmHg, *p* = 0.11) trended to be lower (Figures 6A–C). At the highest equivalent work rate, no differences were found between mMRC ≥1, mMRC = 0 and Con for operating lung volumes and inspiratory reserve volume (Figures 7A,B). Dyspnea relative to VE and dyspnea relative to inspiratory reserve volume with incremental exercise were not different across groups (Figures 7B,C).

### Echocardiography

Results from rest and exercise echocardiography are displayed in Table 5, 6. No differences were found between Long-CoV and Con for right ventricle diastolic area, fractional area change, tricuspid annular plane systolic excursion (TAPSE) and the ratio of peak early mitral inflow velocity to early mitral annular diastolic velocity (E/e’). Pulmonary acceleration time was lower in Long-CoV relative to Con at rest, but similar during exercise. No differences were found between dyspneic-CoV and Con for right ventricle diastolic area or fractional area change, TAPSE, pulmonary acceleration time and E/e’ (left ventricle). Tricuspid regurgitant jet velocity could only be measured in 27% of participants; therefore, pulmonary artery systolic pressure was not calculated.

**TABLE 5:** Rest and exercise (40 W) echocardiography for Long-CoV, no-longer symptomatic (ns)-CoV and Controls. TAPSE; tricuspid annular plane systolic excursion.

Self-reported symptom groupings			Long-CoV	ns-CoV	Con
Heart Rate	bpm	Rest	70 (10)	71 (12)	67 (13)
		40 W	99 (12)	99 (13)	97 (14)
		Delta	29 (8)	28 (9)	30 (8)
Cardiac Output	l/min	Rest	4.7 (1.1)	4.2 (0.8)	4.2 (1.1)
		40 W	7.7 (1.5)	7.1 (1.3)	6.8 (1.1)
		Delta	3.0 (1.2)	2.8 (1.1)	2.6 (0.7)
Right Ventricle Diastolic Area	cm^2^	Rest	20 (2)	18 (3)	18 (4)
		40 W	21 (4)	19 (4)	20 (5)
		Delta	4 (8)	3 (3)	0 (6)
Fractional Area Change	%	Rest	37 (8)	45 (7)	39 (5)
		40 W	43 (6)	45 (7)	41 (7)
		Delta	8 (16)	−2 (9)	0 (12)
TAPSE	mm	Rest	25 (3)	23 (3)	24 (4)
		40 W	29 (5)	28 (2)	28 (4)
		Delta	3 (8)	5 (4)	3 (3)
Pulmonary Acceleration Time	ms	Rest	154 (18)^†^	154 (10)	172 (28)
		40 W	159 (24)	156 (15)	158 (22)
		Delta	4 (18)^†^	2 (12)	−14 (31)
Left Ventricle E/e’ (average)	ratio	Rest	6.4 (1.7)	6.6 (1.4)	6.4 (1.8)
		40 W	7.1 (2.2)	6.7 (2.4)	8.1 (2.4)
		Delta	0.6 (2.6)	−0.1 (1.6)	1.6 (2.1)

†, *p* < 0.05 vs. Con; ‡, *p* < 0.05 vs. ns-CoV.

**TABLE 6:** Rest and exercise (40 W) echocardiography data for mMRC≥1 (dyspneic-CoV), mMRC = 0 and Controls. TAPSE, tricuspid annular plane systolic excursion.

COVID split by mMRC			mMRC≥1	mMRC = 0	Con
Heart Rate	bpm	Rest	73 (11)	69 (11)	69 (14)
		40 W	102 (10)	100 (13)	98 (15)
		Delta	28 (6)	32 (9)	29 (7)
Cardiac Output	l/min	Rest	4.5 (1.3)	4.6 (1.0)	4.1 (1.1)
		40 W	7.1 (1.8)	7.8 (1.4)	6.5 (0.6)
		Delta	1.9 (2.4)	3.1 (1.0)	2.4 (0.6)
Right Ventricle Diastolic Area	cm^2^	Rest	18 (2)	19 (2)	17 (2)
		40 W	19 (5)	20 (5)	19 (3)
		Delta	−2 (7)	3 (3)	2 (2)
Fractional Area Change	%	Rest	37 (10)	39 (7)	40 (6)
		40 W	44 (7)	43 (6)	43 (7)
		Delta	7 (14)	2 (10)	5 (6)
TAPSE	mm	Rest	23 (2)	25 (4)	24 (4)
		40 W	29 (7)	28 (3)	27 (4)
		Delta	5 (7)	1 (8)	3 (2)
Pulmonary Acceleration Time	ms	Rest	153 (20)	157 (13)	166 (28)
		40 W	153 (22)	161 (22)	156 (20)
		Delta	0 (15)	4 (16)	−10 (27)
Left Ventricle E/e’ (average)	ratio	Rest	6.1 (2.0)	6.9 (1.2)	6.5 (1.8)
		40 W	7.8 (3.3)	7.0 (1.1)	8.7 (1.7)
		Delta	1.4 (3.2)	−0.3 (1.2)	2.1 (1.7)

†: *p* < 0.05 vs. Con; ‡: *p* < 0.05 vs. mMRC = 0.

## Discussion

Exercise testing is a valuable tool to evaluate functional capacity (i.e. VO_2peak_) and mechanisms of dyspnea and exercise intolerance (Radtke et al., 2019; Stickland et al., 2022). This study examined exercise responses in a sample of relatively young, non-obese and comorbidity-free COVID-19 participants to determine whether persistent symptoms/dyspnea were associated with altered cardiorespiratory function. In Long-CoV participants, ventilation for a given workload during submaximal exercise was elevated; however, resting cardiopulmonary function, VO_2peak_, and cardiopulmonary responses to exercise were otherwise normal. Similarly, when participants with a history of COVID-19 were grouped according to baseline dyspnea (mMRC grade 0 vs. ≥ 1), we found that dyspneic-CoV participants had greater dyspnea and ventilation for a given workload during submaximal exercise, while resting cardiopulmonary function, VO_2peak_ and cardiopulmonary responses to exercise were normal. COVID-19 participants who report greater baseline dyspnea appear to have greater ventilation during submaximal exercise, which would contribute to an increased perception of exertional dyspnea. Importantly, during submaximal exercise, dyspnea appropriately matched ventilation, and the greater ventilation in dyspneic-CoV participants did not appear to be due to alterations in gas exchange, lung mechanics or any other impairment in cardiopulmonary physiology.

### Pulmonary function

Previous studies have reported pulmonary function patterns consistent with restrictive defects in COVID-19, which normalize over time (Anastasio et al., 2021; Torres-Castro et al., 2021). In contrast to previous studies, the vast majority of participants in the present study were not hospitalized and Long- and dyspneic-CoV participants reported persistent dyspnea in the absence of restrictive defect or other pulmonary function impairment. The results of the present study reinforce findings by Lam et al., which suggest that a PFT may be insufficient to evaluate abnormal dyspnea after COVID-19 (Lam et al., 2021).

### Mechanisms of exertional dyspnea

In obstructive and restrictive lung disease, heart failure (HF) and PH, dyspnea and exercise intolerance can be attributed to; impaired gas exchange, exaggerated ventilatory responses to exercise, dynamic hyperinflation (early critical tidal volume mechanical constraint), and/or elevated pulmonary vascular pressures (Arena and Sietsema, 2011; Guenette et al., 2014; Faisal et al., 2015; Obokata et al., 2018). Accordingly, each of these potential mechanisms of dyspnea were investigated.

### Gas exchange

Given the extensive reporting of arterial hypoxemia and impaired DLCO in COVID-19, impaired gas exchange was a strong candidate mechanism of abnormal exertional dyspnea in Long- and dyspneic-CoV (Torres-Castro et al., 2021). Lerum et al. (2021) reported that 25% of patients hospitalized for COVID-19 had a DLCO below the LLN and ground-glass opacities (CT) 3-months after discharge; however, these abnormalities were not associated with dyspnea, arterial hypoxemia, or a reduction in 6-min walk distance. In the present study, only 2 (7%) COVID-19 participants had an SpO_2_ below 90% at peak exercise, and diffusing capacity (Figures 2C, 5C), D_M_ and V_Cap_ were not different between Long- or dyspneic-CoV and controls (Tables 3, 4). These data indicate that gas exchange abnormalities are not requisite for persistent dyspnea in COVID-19 and do not explain abnormal exertional dyspnea during submaximal exercise in the COVID participants examined in the present study.

### Ventilatory response

Ventilation, as measured at the highest equivalent submaximal workload, was elevated in Long- and dyspneic-CoV as compared to Con. Importantly, the dyspnea-ventilation relationship with incremental exercise was similar between Long- and dyspneic-CoV and controls, indicating that COVID-19 participants do not sense dyspnea differently during exercise relative to controls. Therefore, the greater dyspnea observed during submaximal exercise, but not at rest, in dyspneic-CoV could be explained by an exaggerated ventilatory response to exercise and the associated increased respiratory neural drive (Guenette et al., 2014). It is unclear why ventilation was increased in dyspneic-CoV, as the respiratory pattern does not completely align with other cardio-circulatory and respiratory diseases (Hansen and Wasserman, 1996; Arena et al., 2004; Neder et al., 2016). Dyspneic-CoV participants displayed a trend of mild tachypnea, but maintained normal tidal volume—in contrast to HF, PH and ILD where a rapid, shallow breathing pattern leads to increased anatomical deadspace ventilation and increased VE/VCO_2_ (Ponikowski et al., 2001; Wensel et al., 2013; Neder et al., 2016). Moreover, dyspneic-CoV participants displayed normal DLCO and V_Cap_—contrasting with mild COPD where pulmonary vascular dysfunction marked by reduced DLCO and V_Cap_ leads to increased alveolar deadspace ventilation and increased VE/VCO_2_ (Tedjasaputra et al., 2018; Phillips et al., 2021). Finally, hyperventilation is observed across cardio-circulatory and respiratory diseases which can be secondary to muscle afferent feedback and deconditioning (Hansen and Wasserman, 1996; Arena et al., 2004; Neder et al., 2016). Peripheral deconditioning leading to an exaggerated ventilatory response to exercise is supported by a case series of eight dyspneic COVID-19 patients, 3-months after mild COVID-19 (Motiejunaite et al., 2021). In these patients, PFTs were normal, VE/VCO_2_ was elevated, and no patients reached their predicted VO_2peak_, which could be explained by greater peripheral fatigue (Motiejunaite et al., 2021). Our results extend and contrast these findings by reporting a similarly exaggerated ventilatory response to exercise, exertional dyspnea, and a trend of reduced ventilatory efficiency, but in the absence of VO_2peak_ impairment, suggesting that peripheral deconditioning is an unlikely explanation for increased ventilation during submaximal exercise.

### Operating lung volumes

Airway obstruction leading to dynamic hyperinflation and critical mechanical constraint is a well-known mechanism of dyspnea in COPD (O'Donnell et al., 2017). Long- and dyspneic-CoV participants did not demonstrate dynamic hyperinflation nor infringement on inspiratory reserve volume, as tidal volume was augmented appropriately, and end-expiratory and end-inspiratory lung volumes responded appropriately to exercise (Figures 4A, 7A). Thus, operating lung volume responses were normal and would not explain abnormal exertional dyspnea in the COVID participants examined in the current study.

### Pulmonary vascular pressure

In contrast to previous reports, we did not identify structural or functional cardiac changes in Long- or dyspneic-CoV (Szekely et al., 2020). Aspects of the exercise response in dyspneic-CoV such as trends for tachypnea and hyperventilation are similar to what would be observed in conditions such as PH, but indicators of pulmonary vascular pressure (pulmonary acceleration time, E/e’) in the present sample were not elevated at rest or during exercise (Laveneziana et al., 2013). Moreover, metrics of right ventricular function (TAPSE, fractional area change) were not impaired in dyspneic or Long-CoV (Tables 5, 6). Taken together, these data imply that pulmonary vascular pressures and cardiac physiology are unlikely to contribute to persistent dyspnea in our Long- or dyspneic-CoV participants.

Caution is warranted when interpreting findings of increased ventilation and dyspnea during submaximal exercise. As increased ventilation in dyspneic-CoV does not appear to be a compensatory mechanism to maintain adequate gas exchange and the ventilation-dyspnea relationship was preserved, findings may partially be due to the fitness discrepancy, possibly predating COVID-19 infection. However, at lower work rates (40, 60 W) which more closely approximate the metabolic cost of walking up a slight hill (i.e. mMRC = 1), differences in submaximal exercise metrics were not detectable. This reinforces that factors beyond cardiopulmonary physiology appear to contribute to perceived symptom burden and exertional dyspnea after COVID-19.

## Limitations

A limitation of the study is the cross-sectional design. VO_2peak_ was lower in Long- and dyspneic-CoV relative to Con; however, we believe that this is due to selection bias, whereby exceptionally healthy participants volunteered for control groups, while symptomatic participants were recruited from Long-COVID clinics. However, we cannot rule out the possibility of a COVID-19-related drop in cardiopulmonary fitness. Given that pulmonary dysfunction and cardiopulmonary abnormalities were not observed in Long- or dyspneic-CoV, and that VO_2peak_ in Long- and dyspneic-CoV was ∼106 and 98% of age-predicted normative data, respectively, a reduction in VO_2peak_ as a result of COVID-19 infection is unlikely.

The results of this study should be extended with caution, particularly to those with severe initial infection and COVID-19 pneumonia requiring hospitalization. Only a small proportion of the sample included in the study were hospitalized during acute infection. Evidence suggests that after pneumonia and hospitalization, symptoms and functional impairment may persist for 6-months or longer, and that some symptoms may be attributable to underlying comorbidities (Metlay et al., 1997; Fumagalli et al., 2021).

Our sample consisted of a female to male ratio of ∼4:1 reporting Long-CoV and baseline dyspnea. The high female prevalence was consistent with our Long-CoV clinic, and others have reported female sex as a risk factor for Long-CoV (Sudre et al., 2021). More research is needed as it remains unclear whether potential sex differences in immune response, cardiopulmonary physiology, or other factors, such as care seeking behaviors, explain the female predominance of Long-CoV (Sudre et al., 2021).

Finally, emerging evidence suggests a potential role of autonomic dysfunction in cardiopulmonary impairment after COVID-19 (Shouman et al., 2021; Stute et al., 2021). Shouman et al. (2021) report impaired heart rate and blood pressure responses to physiologic stress, and orthostatic intolerance temporally related to COVID-19 infection that does not differ compared to orthostatic intolerance encountered following other viral disorders. Autonomic function was not measured in the present study, however, cardiopulmonary responses to exercise (including heart rate and blood pressure) and peak exercise capacity were normal in Long- and dyspneic-CoV.

Carotid chemoreceptor activity/sensitivity can play a role in cardiorespiratory regulation during exercise (Byers et al., 2019). We did not assess chemoreceptor activity/sensitivity in the present study; however, P_ET_CO_2_ was not different across groups during exercise (Figures 3B, 6B), suggesting similar ventilatory responses and carotid chemoreceptor sensitivity across groups. Taken together, these data suggest that while autonomic function may be impaired in isolated Long-CoV cases, autonomic dysfunction is unlikely to explain elevated exertional dyspnea after COVID-19 in our sample.

## Conclusion

The high incidence of Long-CoV, even after a non-severe COVID-19 infection, imposes a monumental healthcare burden as patients seek care for persistent dyspnea and other symptoms. Our data provide insight into persistent dyspnea after COVID-19 by demonstrating that in young, non-obese and comorbidity-free COVID-19 participants, dyspnea is not due to overt cardiopulmonary impairment or exercise intolerance, and that dyspnea sensation pathways are normal. Factors beyond cardiopulmonary physiology likely contribute to symptom burden, indicating that interventions focusing on dyspnea management may be appropriate for the phenotype of Long-CoV patients who report dyspnea.

## Data Availability

The raw data supporting the conclusion of this article will be made available by the authors, without undue reservation.

## Abbreviations

ANOVA, Analysis of variance; BMI, Body mass index; Con, Control group or participants; COPD, Chronic obstructive pulmonary disease; COVID-19, Corona Virus Disease 2019; Long-CoV, persistently symptomatic COVID-19; ns-CoV, no-longer symptomatic COVID-19; CT, Computed tomography; DLCO, Lung diffusing capacity for carbon monoxide; DM, Lung diffusing membrane capacity; E/e’, Ratio of early mitral filling velocity to early mitral annular tissue velocity; EQ-5D, EuroQoL 5-dimension; FEV1, Forced expiratory volume in 1-s; FiO_2_, Fraction of inspired oxygen; FVC, Forced vital capacity; HF, Heart failure; ILD, Interstitial lung disease; LLN, Lower limit of normal; mMRC, Modified Medical Research Council dyspnea grade; PCFS, Post-COVID functional scale; PETCO2, Pressure of end-tidal carbon dioxide; PFT, Pulmonary function test; PH, Pulmonary hypertension; TAPSE, Tricuspid annular plane systolic excursion; TLC, Total lung capacity; VCap, Pulmonary capillary blood volume; VE, Minute ventilation; VE/VCO_2_, Ventilation with respect to carbon dioxide production; VO_2_peak, peak oxygen uptake; W, Watts.
